# An Experimental Inquiry into the Nature, Cause, and Varieties, of the Arterial Pulse; and into Certain Other Properties of the Larger Arteries, in Animals with Warm Blood; Illustrated by Engravings

**Published:** 1816-07

**Authors:** 


### PART II.

### COMPREHENSIVE ANALYTICAL REVIEW OF MEDICAL LITERATURE.

"Tros, tyriusve, *nobis* nullo discrimine agetur."

*An Experimental Inquiry into the Nature, Cause, and Varieties, of the Arterial Pulse; and into certain other Properties of the larger Arteries, in Animals with warm Blood; illustrated by Engravings.* By CALEB HILLIER PARRY, M. D. F. R. S. &c. &c.

The veneration in which the name of Parry has long been held by us, was not diminished by the perusal of his late work, the Elements of Pathology; and therefore we took up the present volume with anticipations of pleasure and profit. The nature of the subject, however, though at all times interesting, was so obscure, and hitherto unsatisfactorily explained, that we did not make up our minds for so gratifying a treat as the preceding labours of Dr. Parry had afforded us. Nevertheless, we perused page after page till the end, with mingled emotions of satisfaction, but unmixed sentiments of respect.

The work opens with a synthetical view of the experiments on which the subsequent deductions of the essay are founded. The experiments were made on different animals, and on different arteries of animals. They all tended to prove one leading fact, that arteries suffered no dilatation or contraction during the systole or diastole of the heart. We shall introduce one of the experiments as an example.

*September* 22, 1814. "In a ram, both carotid arteries were laid bare by Mr. George Norman, in the presence of Mr. Coombs, two others of his pupils, and myself.

"Notwithstanding the animal was greatly agitated, there was not in either carotid the least appearance of dilatation during the systole of the ventricle, or contraction during its diastole: nor except when the animal breathed, was there any degree of loco-motion perceived in these arteries, which remained completely quiet and at rest. Nevertheless, when either artery was compressed between the finger and thumb, the pulse was very strong and distinct.

"Both arteries were adequately tied by Mr. Norman, each with a single ligature; after which, the ram stood and walked about well, apparently suffering little inconvenience." p. 2.

A great variety of experiments are afterwards detailed; but all tending to establish the fact, that no actual dilatation or contraction takes place in the arterial system from the action of the heart.

We shall pass over the chapters on the structure, and on the power of arteries very cursorily. Dr. Parry's reasonings and experiments on the relative proportions and power of tonicity and elasticity in arteries, are, as usual, acute and accurate. They lead to conclusions very different from those of preceding physiologists, and more especially of Mr. Hunter.

That gentleman considers

"The natural pervious state of an artery, to be that to which the elastic power naturally brings a vessel, which has been stretched beyond, or contracted within, the extent which it held in a state of rest. The stretched is that state produced by the impulse of the blood in consequence of the contraction of the heart; from which it is again brought back to the natural state by the elastic power, perhaps assisted by the muscular." *On the Blood.* p. 116.

From this and some other passages, Mr. Hunter appears to think that the natural state of an artery during life is that to which the elastic power spontaneously brings it, when no longer forcibly dilated by the systole of the heart. But Dr. Parry's experiments tend to prove, on the contrary, "that during health the larger arteries of a living animal, as well under the diastole as the systole of the left ventricle, are in a state of distention, to which they are forcibly impelled by their contained blood, against their mechanical power of elasticity." p. 70.

In order to elucidate this point, Dr. Parry caused two ligatures, an inch and a half distant, to be made at the same instant on the carotid of a living sheep. The artery, measured half way between the ligatures was $\frac{7}{4}$ of an inch in circumference. The blood being suffered to flow out of this included part, from a lancet puncture, the artery, in the same spot as before, measured in circumference only $\frac{160}{400}$ of an inch. Hence the circumference of the artery was immediately reduced $\frac{40}{400}$ of an inch by the mere evacuation of the blood contained in the artery. In a horse, the circumference of the right carotid artery was $\frac{316}{400}$ of an inch. It was firmly tied at some distance before the part measured, and a second ligature was applied an inch beyond the first. In an hour, the intermediate portion was punctured. The blood having coagulated in the artery, little more than serum flowed out. The vessel being now measured in the same place as before, was found to be only $\frac{252}{400}$ of an inch in circumference. In sixteen hours after the death of the animal by bleeding from the left carotid, the circumference of the artery at the same point as before, was $\frac{379}{400}$ of an inch. Hence, the *elasticity* was as $\frac{137}{400}$ of the circumference of the artery, and the *tonicity* as $\frac{127}{400}$.

"From these two last Experiments, says Dr. P. it appears, that the forced distention of the artery does not necessarily depend on the impulse of the blood from the systole of the ventricle, since it existed when the communication with the stream of blood from the heart was interrupted by a ligature.

"As during the common circumstances of health, arteries are naturally distended beyond that degree, to which they would spontaneously contract by their elastic power, so they are capable of suffering a still greater degree of distention, from increased fulness of blood in the general system, or from those excessive determinations of blood to particular parts, which occur, on various occasions, in the animal frame. Thus it is generally admitted, that, if the passage of the blood through one arterial trunk is intercepted, the collateral branches, supplying the part, undergo an increased distention, in order fully or partly to compensate the loss.

"This conclusion was rendered very probable by what occurred in the ram, Experiment 15; in which one carotid had been tied nearly eleven months before, and the other was found much larger than any that we had before observed.

"In order, however, more accurately to investigate this point, the right carotid of a small ewe having been found to be in circumference $\frac{233}{400}$ of an inch, the left carotid was tied; a few minutes after which, the right had gained in circumference $\frac{24}{400}$ of an inch.

"In another ewe, the right carotid being $\frac{222}{400}$ of an inch in circumference, was again measured twelve minutes after the left had been tied, and was found to have gained in circumference $\frac{98}{400}$ of an inch.

"I know of no actual measurements to prove that a similar effect takes place, in inflammation and other maladies, in the larger arteries supplying the parts so affected; but we may reasonably presume its existence from the tangible, and sometimes visible, increase of size in the artery, as well as from the great perceptible enlargement of the veins leading from the diseased parts.

"From various preceding considerations, it is natural *à priori* to conclude, that, when under the state not only of the usual healthy dilatation of arteries, but of that increased dilatation which often accompanies disease, the distending cause, which is a certain quantity or momentum of blood, is diminished, the elasticity will tend to contract them, so as, within certain limits, to accomodate them to the quantity of blood which they ought to convey. If, however, the elasticity should be inadequate to the requisite force or degree of contraction, the tonicity, or vital power, may assume the office of contraction where it was left by the elasticity, and carry it to the necessary extent.

"In order to ascertain how far this accommodation of arteries to the loss of blood from the system would reach, I suggested the Experiment 24; in which, the circumference of the right carotid in a ewe having been first raised, by a ligature on the left, from $\frac{222}{400}$ of an inch, to $\frac{320}{400}$, was successively diminished to $\frac{228}{450}$, $\frac{250}{400}$, $\frac{238}{400}$, $\frac{235}{400}$, $\frac{222}{400}$, $\frac{201}{400}$, $\frac{191}{400}$, and $\frac{161}{400}$ of an inch, by as many bloodlettings of eight ounces each, from the left jugular vein, till the death of the animal.

"So also in Experiment 27, the right carotid of another ewe, which was $\frac{222}{400}$ of an inch in circumference, was successively reduced to $\frac{196}{400}$, $\frac{178}{400}$, $\frac{170}{400}$, $\frac{145}{400}$, and $\frac{127}{400}$, of an inch, by similar bleedings from the same vein.

"Hence we may understand, how readily the arteries may become more or less capacious, in proportion to the quantities of blood which may either exist in the whole system, or may be determined to particular parts."

Dr. Parry thinks it probable, however, that the relative capacities of entire arteries, or certain parts of them, may be affected by other agents than the blood contained in them; as, for instance, from the mere exposure of arteries, some examples of which are given. From these and other Experiments, it appears, that the fibres of the middle coats of arteries have no power of elongation, like the radiated fibres of the iris, but merely of contraction, and that in dilatation they are passive. In respect to elasticity, it is obvious, that as arteries are already dilated beyond the degree which that agent, if not counteracted, would have permitted, any increase of dilatation must arise, not from an augmentation, but a diminution of the action of that power; hence we may conclude, that the cause of the common dilatation of the larger arteries, and still more of that which is preternatural, is the mechanical distension of the blood. p. 78.

THE NATURE AND CAUSE OF THE ARTERIAL PULSE.

Physiologists are not yet agreed respecting the cause of the pulse. Haller plainly attributes it to the alternate dilatation and contraction of the artery from the impulse of the wave of blood, corresponding with the systole and diastole of the left ventricle of the heart. "D'abord je me suis assuré que le sang, poussé par le cœur, dilate les arteres, et forme ce battement, qu'on appelle le pouls." *Memoires sur le mouvement du Sang.* p. 33. He acknowledges, however, that frequently in living animals examined by dissection, no pulse, nor any dilatation of the artery, can be seen.

Bichat, finding that in denuded arteries there was no dilatation during the systole of the left ventricle, considered the pulse as chiefly owing to a kind of locomotion in the whole artery, wherein it springs against the finger in the abovementioned state of the ventricle.

"To illustrate the term *locomotion*, says Dr. P. let us suppose a piece of flexible cane, which we may move up and down in the direction of its length—may cause to advance or recede—may bend in various ways, or may even turn on its own axis, while at the same time, its diameter or size is in no respect changed." 92.

DUMAS, maintains with GALEN, that the dilatation and contraction of arteries are inherent in themselves, and independent of the mechanical impulse from the heart.

RICHERAND, with the greater number of preceding physiologists, attribute the pulse to an increase of diameter in the artery from ventricular impulse of blood behind. PORTAL is of the same opinion. Sœmmering contends for a similar explanation of the pulse, and the writers of our own country are universally agreed that the dilatation and contraction of the arteries depend on the systole and diastole of the left ventricle.

The question is not purely of philosophical curiosity, since it involves a point of consequence in the practice of modern surgery. Were a young surgeon taught to expect that he should be able to distinguish by dilatation and contraction, an artery which he was required to lay bare, and find this test to be wholly wanting, his embarrasment might be considerable; and were a more experienced operator about to tie a deep seated artery, and find himself unable to discover that artery by the touch, or having made his ligature, remain ignorant whether it was rightly applied till assured by the event, his anxiety may be readily conceived.

" In the course of our experiments, says Dr. P. in which a carotid, separated from all its attachments, was fully exposed to view, and yet no movement was observable in it, we were sometimes equally surprized to find, that, when the finger was pressed against it, or even when it was removed out of its place by the force of the finger beneath it, or on one side, it was equally void of all pulse to the touch.

" A frequent repetition of the same results led to a discovery of what appears to be the nature of the pulse itself.

" The blood, in every part of the arterial system, from the mitral valves, outwards through the whole frame, to the right auricle, may be considered as a set of continuous columns, possessing little compressibility, and filling the tubes in which they are contained.

" When, by the contraction of the left ventricle, the blood included in it is forcibly expelled into the aorta, all these columns receive the shock of propulsion at the same instant. But the velocity, during this systole being greater than during the diastole, the momentum, and consequently the impulse, in every direction, is also greatest in the systole. When, therefore, an artery is compressed with the fingers, in the usual mode of feeling the pulse, the blood, in consequence of the systole, rushing into the artery with an increase of momentum, gives a stronger impulse of dilatation to the fingers, than from the less momentum which exists during the diastole, and thus produces the phænomenon of pulse.

" Hence, it appears, that the pulse is the effect, not of an extension of an artery beyond its usual diameter, but of a stronger effort, during the systole of the ventricle, than during its diastole, to restore the usual diameter of the artery, which had been diminished by compression.

" Since, also, the excess of velocity from the systole extends throughout the whole of the space compressed by the fingers, it is evident that the distending effort, producing the sense of pulsation, must also be felt throughout that space.

" Before this explanation could be fully admitted, it was necessary to ascertain the cause of the converse of this state; or the reason why a pulse was sometimes wanting in an artery exposed to view; and susceptible of any mode of examination by the touch. Reiterated trials demonstrated, that this deficiency was owing to the following circumstances. The coats of the carotids are so firm, that when either impelled against any soft substance, or simply moved out of their place, these arteries readily recede; suffering no reduction of diameter, and, therefore, giving no sensation of a pulse. But if they are confined by any hard substance placed behind them, so as to resist a change of position from the pressure of the finger, or if they are squeezed between the finger and thumb, so as, in either case, to suffer a certain reduction of diameter, then the pulse never fails to exhibit itself.

"These latter experiments, therefore, while they show that the pulse does not exist under the mere contact of the finger with the artery, and, therefore, completely refute the supposed dilatation of an artery by the systole of the ventricle, as an object of touch; by ascertaining the precise circumstances under which a pulse does or does not exist, demonstrate, beyond all reasonable objection, the nature of that phænomenon.

"Hence we see the probable reason, why no pulse is said to have been discovered in those surgical operations, to which I have before alluded. If, however, the principle as to both states be well established, we have only sufficiently to diminish with the finger the diameter of an artery; and then, if it be of a certain size, and the course of blood through it, from the heart, be uninterrupted, we shall not fail to feel the pulse in that artery."

From these facts, Dr. Parry explains a phænomenon which was a stumbling block in the way of Mr. Hunter and other physiologists; viz. why a *denuded* artery exhibits no pulsation, while the latter is visible when the artery is surrounded with its natural coverings.

In order to discover this cause, says he, lay bare an artery, and no dilatation is perceptible; but place behind it any hard substance that prevents its recession, and then the finger placed opposite to this on the artery, immediately feels a propulsive stroke at each systole of the left ventricle. In short, the whole which is necessary is, that the artery should have its diameter in a certain degree reduced; and then the substance reducing it, if not too ponderous, will be driven off in leaps, always according with the systoles of the heart, and often perceptible to the eye.

"In this way the pulsation of arteries may be frequently seen, when they are unusually compressed by muscles, or other intervening substances. Hence fæces in the colon; or other indurations in the abdomen, near, or in close contact with the aorta, by compressing that vessel, often both to the eye and touch, simulate aneurisms; and hence, aneurisms themselves, forming for the blood, channels which are preternaturally compressed in various directions, suffer jerks from the impulse of the ventricle, which often shake all the neighbouring parts.

"It is not then, the mere intervention between the eye or finger, and an artery, of any substances, whether hard or soft, that, conformably to the opinion of Mr. Hunter, makes its pulsations more sensible; but it is because the substances thus intervening, often interrupt the current of blood through the artery, and therefore receive a shock, which may be tangible or visible, from each systole of the ventricle." 118

In our review of Dr. Carson's work, we expressed our conviction that the heart possessed the power of *expanding* its cavities, as well as of *contracting* them; and, consequently that a suction influence was exerted on the venous system. It appears, that Dr. Parry is of a similar opinion.

"Those, says he, who maintain that the pulse is owing to the alternate dilatation and contraction of arteries, from the systole and diastole of the left ventricle, have probably deduced their conclusion from the analogy of the action of the heart itself. In this organ, they presume that each cavity is dilated in consequence of the distending power of the blood impelled into it, and then contracts itself in consequence of that distention. To me, however, it appears that this theory, however specious, is absolutely erroneous. The different compartments of the heart are so far from expanding, in consequence of the blood which is driven into them, that, *when altogether empty of blood, and even separated from the animal, they expand in a greater degree than when in their natural situation during life and healthy circulation.* Hence, Mr. Hunter himself, notwithstanding the view which he gives of the action of the heart, acknowledges that this organ, after death, has a larger volume than while the animal is living." p. 122.

Now if the chambers of the heart are capable of dilating themselves, they must as inevitably *suck* the blood from the venous system, as the expansion of a pair of bellows draws in air through the pipe. There does not, therefore, appear any necessity for the *resiliency* of the lungs, either in the fœtal or subsequent state, though in the latter it is not impossible that it may have some operative effect.

In Dr. Parry's remarks on anomalies of the pulse, some curious instances are related, shewing the accuracy of attention which our author must have long been in the habit of bestowing on every form and phænomenon of disease. Dr. Parry is not an every day physician; he has not driven from house to house, making a few common place enquiries of the patient, and writing a long prescription which the friends of the sick must see, in order to give them a great idea of his abilities; he is a man of no ordinary stamp, as every part of his writings most emphatically evinces.

"Twenty instances, says Dr. P. have occurred to me of the total loss of pulse in the radial arteries, from various maladies of the *alimentary canal,* whether a considerable time before death, or under circumstances that admitted of recovery; while at the same time the pulse in the carotids was full and strong. In one instance of general dropsy, the patient had no pulse in either wrist for seventeen days; yet was restored to perfect health. Sometimes, before death, the pulse has been wanting in one radial only.

"I have seen a total loss of pulse in one arm, with coldness, but complete power of motion in that part; while the other arm was warm, and possessed a perfectly good pulse, but had lost all power of voluntary motion. These symptoms commenced suddenly, two or three days after parturition. The patient soon died, but a dissection was not obtained.

"In another case, a young man, labouring under pulmonary hectic, was found to have lost the pulse in one wrist, immediately after coming out of a warm bath. Several months afterwards, it had returned, though in an almost imperceptible degree.

"Another patient, a female of middle age, the mother of several children, affected with severe cough, was, apparently, in a state of convalescence, and walking about her house; when it was discovered that the pulse in one arm was wholly wanting. A few days afterwards, she died suddenly. The whole course of the aorta was carefully examined; but no deviation from the healthy state could be perceived in it." 140.

The limits of our analysis will not permit us to extract any more from Dr. Parry's work; but we shall make a few reflections which suggested themselves during the perusal of it. We entertain not the slightest doubt of the accuracy of the experiments made by Dr. Parry and his friends, and yet we must beg leave to dissent from the apparently irresistible conclusions which he draws. That the calibre of an artery undergoes an alternate dilatation and contraction, or increase and diminution, during the systole and diastole of the heart, we still believe, notwithstanding the numerous experiments which seem to prove the contrary;—and for the following reasons. 1st. We cannot conceive how a jet of fluid can be forcibly thrown into a *full tube* (as Dr. P. maintains the aorta or other arteries to be) without an impulse being made against the sides of that tube, either by the new fluid, or that previously contained in the tube. And the tube being manifestly dilatable, it follows that this impulse must, in some degree, alter the calibre of the vessel. Secondly, from the numerous opportunities which we have had of seeing arteries bleed, we are convinced that the current of blood during the diastole of the heart, though not so rapid as in the systole, is still rapid and strong. Now if the jet of blood from the left ventricle merely displaced so many inches (say three) from the root of the aorta, impelling what was there before, forward along the artery, *without altering its dimensions,* the last jet must remain in perfect quiescence during the diastole of the heart; for it is evident that it cannot continue to run, however slowly, without the artery closing upon it behind, otherwise a vacuum would be left at the root of the aorta, which is impossible. The same reasoning will apply to every subsequent space along which the current is impelled; for it is absolutely impossible that a fluid can continue to flow through a tube, while there is no fluid entering that tube, nor contraction of the tube itself taking place; which must be the case during the diastole of the heart, if the arteries remain the same. According to Dr. Parry's theory then, the blood must go through the arterial and capillary system by jets during the systole, and remain completely quiescent during the diastole; but this Dr. P. cannot possibly mean. We request him, therefore, to prove to us how a current can continue to run through a tube during the time that no fluid is entering the tube, unless the calibre of that tube be lessened at the same time?

Again, were the power of the heart sufficient to keep up a flow of blood through the arteries, both during the systole and diastole, and these arteries to remain passive tubes, of what use are the valves at the origin of the aorta, for what other power than that of the contraction of the arteries could turn back the flow of blood towards the ventricle, against which nature seems to have so carefully provided by the semilunar valves?

We can very easily conceive that no visible dilatation of an artery at any distance from the heart, could be discovered in Dr. Parry's experiments. If we reflect on the very small proportion of *additional* blood which a single systole of the heart can distribute to any one artery; and on the lightning-like rapidity with which the ring of dilatation shoots from the origin to the termination of the artery, we may readily believe that any attempt to *measure* so minute and so fugitive a dilatation would be utterly impracticable.

Allowing that the left ventricle of the heart dilated five or six inches in length of the arch of the aorta, at each systole, and that this dilatation amounted to a sixth, or even a fourth of an inch diametrically; such would be the diminution of these in the carotid artery, and the amazing velocity with which the dilated ring flew along the tube, that any admeasurement of it would be altogether impossible.

On the other hand, granting that the whole arterial system be full, as no doubt it is, at the commencement of each systole, and that the ventricle forcibly injects two or three ounces of blood into that system, the rise or dilatation being instantaneous and simultaneous throughout the whole tree of arteries; how very minute must that dilatation be; and how incapable of measurement or calculation?

Upon the whole, we still firmly believe that at each systole of the heart, there is either a rapid vermicular, or a general and consequently very minute, dilatation of the arterial system; and that on no other supposition can the continued flow of blood during the diastole of the heart be accounted for. Nay, we assert that it is impossible for the blood to *continue* to flow through *passive* tubes, during the period that no blood is entering the origin of these tubes, as in the diastole of the heart, when in reality the head of the column is hermetically sealed. To be convinced of this, let a fluid be propelled through any passive tube of iron or glass, with ever so great a velocity, and the moment that we close *either end* of the tube, the current is arrested completely. How then can the blood flow through the arteries when their origin, the root of the aorta, is closed in the diastole? Only indeed by the reaction of the arteries themselves.

---

